# An International Delphi Study on Barriers to On‐Demand Treatment of Hereditary Angioedema Attacks

**DOI:** 10.1002/clt2.70159

**Published:** 2026-03-04

**Authors:** Aleena Banerji, Emel Aygören‐Pürsün, Noemi‐Anna Bara, Jonathan A. Bernstein, Stephen Betschel, Laurence Bouillet, Paula J. Busse, Teresa Caballero, Mauro Cancian, Danny M. Cohn, Timothy Craig, Henriette Farkas, Anete Sevciovic Grumach, Michihiro Hide, Sorena Kiani‐Alikhan, Hilary J. Longhurst, William R. Lumry, Marc A. Riedl, Marcin Stobiecki, Anna Valerieva, Andrea Zanichelli

**Affiliations:** ^1^ Division of Rheumatology, Allergy and Immunology Massachusetts General Hospital Boston Massachusetts USA; ^2^ Universitätsklinikum Frankfurt Frankfurt Germany; ^3^ Romanian Hereditary Angioedema Expertise Centre Centrul Clinic Mediquest Sangeorgiu de Mures Romania; ^4^ Department of Internal Medicine, Division of Rheumatology, Allergy and Immunology University of Cincinnati College of Medicine Cincinnati Ohio USA; ^5^ Department of Medicine St. Michael's Hospital, University of Toronto Toronto Ontario Canada; ^6^ Internal Medicine Department National Reference Center of Angioedema CREAK, Grenoble University Hospital Grenoble France; ^7^ Division of Allergy and Clinical Immunology Icahn School of Medicine at Mount Sinai New York New York New York USA; ^8^ Department of Allergy La Paz University Hospital, Hospital La Paz Institute for Health Research (IdiPAZ—Group 44), Biomedical Research Network on Rare Diseases (CIBERER U754) Madrid Spain; ^9^ Departmental Unit of Allergology University Hospital of Padua Padua Italy; ^10^ Department of Vascular Medicine, Amsterdam Cardiovascular Sciences, Amsterdam UMC University of Amsterdam Amsterdam the Netherlands; ^11^ Department of Medicine Pediatrics MFM and BioMedical Sciences Penn State University Hershey Pennsylvania USA; ^12^ Vinmec International Hospital and Professor of Medical Sciences VinUniversity Hanoi Vietnam; ^13^ Hungarian Angioedema Center of Reference and Excellence, Department of Internal Medicine and Haematology Semmelweis University Budapest Hungary; ^14^ Clinical Immunology Centro Universitário Faculdade de Medicina do ABC (CEUFMABC) Santo André São Paulo Brazil; ^15^ Department of Dermatology Hiroshima City Hiroshima Citizens Hospital Hiroshima Hiroshima Japan; ^16^ Department of Dermatology Hiroshima University Hospital Hiroshima Japan; ^17^ Department of Immunology Royal Free London NHS Foundation Trust London UK; ^18^ Department of Medicine University of Auckland and Department of Immunology, Auckland City Hospital Auckland New Zealand; ^19^ AARA Research Center Dallas Texas USA; ^20^ Division of Allergy and Immunology University of California San Diego California USA; ^21^ Department of Clinical and Environmental Allergology Jagiellonian University Medical College Krakow Poland; ^22^ Department of Allergology Medical University of Sofia, Clinic of Allergology, University Hospital Sofia Bulgaria; ^23^ Department of Biomedical Sciences for Health University of Milan Milan Italy; ^24^ Operative Unit of Medicine, Angioedema Center IRCCS Policlinico San Donato Milan Italy

**Keywords:** Delphi, expert consensus, hereditary angioedema, on‐demand treatment, treatment guidelines

## Abstract

**Background:**

Hereditary angioedema (HAE) is a rare inherited disorder characterized by unpredictable and potentially life‐threatening attacks of swelling. This international Delphi panel aimed to address questions related to on‐demand treatment of HAE attacks.

**Methods:**

A modified Delphi method was conducted with three rounds of surveys. Two non‐voting co‐chairs designed and managed the surveys, data collection, and analysis with a third‐party administrator. The international panel consisted of 19 expert HAE clinicians. Consensus was defined as ≥ 75% agreement with ≥ 75% of panelists voting.

**Results:**

The panel confirmed 24 statements across five key areas related to on‐demand treatment: defining “early” treatment, barriers to early administration, burden of treatment, tolerability and convenience, and patient–clinician interactions. Panelists defined early treatment as ≤ 60 min after onset of an HAE attack. Obstacles to early treatment include recognition of an HAE attack, and embarrassment/anxiety about administering parenteral treatment. Access to on‐demand treatment (i.e., carrying medication, cost, insurance coverage, regulatory approval) can be a burden for patients with HAE, and increasing access may improve adherence to guidelines. Logistical obstacles of parenteral administration that impact convenience, tolerability concerns (e.g., side effects), and cost of medication can all limit early use of on‐demand treatment. Additional options for on‐demand therapies beyond parenteral treatments could reduce some of the burdens. Panelists agreed that patient–physician shared decision‐making should be utilized.

**Conclusions:**

The Delphi consensus statements demonstrate the need for accessible and convenient on‐demand treatments for HAE attacks that will enable patients with HAE to improve adherence to guidelines.

## 1 Introduction

### 1.1 Hereditary Angioedema

Hereditary angioedema (HAE) is a rare inherited disorder that is estimated to affect approximately two per 100,000 patients in the United States and approximately 120,000 globally [1, 2]. Of these patients, most (i.e., approximately 75%–80%) have HAE resulting from C1 inhibitor protein (C1INH) deficiency in the plasma (HAE‐C1INH‐type 1) [3]. Other types are HAE‐C1INH‐type 2, resulting from dysfunction in existing C1INH, and HAE with normal C1INH levels (HAE‐nC1INH); the epidemiology of HAE‐nC1INH is not well‐known [2, 4, 5]. HAE is characterized by recurrent and unpredictable attacks of swelling that impact cutaneous and submucosal tissues and can cause pain, impaired function, and be life‐threatening and potentially fatal when involving the upper airway [4, 6, 7].

### 1.2 Approved and Investigational Therapies for HAE Attacks

Treatment for HAE falls into three main categories: long‐term prophylaxis, short‐term prophylaxis, and on‐demand [7, 8, 9]. Patients can experience HAE attacks despite use of prophylaxis, and when these attacks occur, they should be treated using on‐demand therapy to minimize symptoms and risks [3, 8, 9]. Across multiple regions, there were five approved on‐demand treatments for HAE attacks at the time this Delphi process was conducted in Spring 2025. Berinert (CSL Behring) and Ruconest (Pharming) are plasma‐derived C1INH and recombinant human C1INH, respectively, which are administered intravenously and act by increasing plasma C1INH levels [10, 11, 12, 13]. Cinryze (Takeda) is plasma‐derived C1INH administered intravenously and approved as an on‐demand therapy in the European Union [14, 15]. Ecallantide (Kalbitor, Takeda) is approved as an on‐demand therapy in the United States [16, 17], though not in the European Union, due to safety concerns [18]. It is administered subcutaneously and is an inhibitor of plasma kallikrein, thereby inhibiting conversion of high molecular weight kininogen to bradykinin [16, 17]. Finally, icatibant (Firazyr, Takeda) is a bradykinin B2 antagonist administered by subcutaneous injection [19, 20]. Sebetralstat (EKTERLY, KalVista Pharmaceuticals), an oral plasma kallikrein inhibitor, approved as an on‐demand treatment in the United States, Europe, Japan, and other countries [21, 22, 23, 24], was under investigation at the time of this Delphi consensus study (which concluded in May 2025). The oral bradykinin B2 receptor antagonist deucrictibant (Pharvaris) was also under investigation at the time of the Delphi study [25].

### 1.3 Evolution of Guidelines

Guidelines and recommendations by HAE experts for the treatment of HAE attacks have evolved with increasing clinical evidence [26, 27]. Because of the rare nature of HAE, controlled clinical data have been sparse, treatment recommendations only date back to the early 2000s, and guidelines focus on HAE‐C1INH‐type 1 and type 2. Initial consensus statements prioritized treatment based on the location and severity of the attack. With an expanding treatment landscape and improved knowledge about the impact and burden of HAE attacks, recommendations and guidelines have shifted over time to focus on the early treatment of all HAE attacks and emphasize clinical goals rather than the location of the attack [26, 27].

### 1.4 Current Guidelines

Evaluation of guidelines from professional organizations focusing on HAE (Supporting Information S1: Table 1) found several common recommendations for optimization of on‐demand treatment for HAE attacks [8, 9, 26, 28, 29, 30, 31, 32, 33, 34, 35]. Patients should be educated to treat all HAE attacks, regardless of location or severity, and encouraged to treat HAE attacks early to slow or stop the progression of swelling and shorten the attack. Patients should be educated on self‐administering on‐demand treatment for HAE attacks, except when treating with ecallantide [16], which has a 3% risk of anaphylaxis, and in the United States requires administration by a healthcare professional with appropriate medical support to manage anaphylaxis and HAE. Additionally, in Japan, Berinert P (plasma‐derived C1INH) is only licensed for administration by a healthcare professional [36]. Patients should have access to at least two doses of on‐demand medication [8, 9, 26, 28, 29, 31, 32, 33, 34].

### 1.5 Delphi Goals

While existing guidelines are valuable in providing suggestions and advice for treatment and patient education of this rare disease, there are outstanding questions surrounding on‐demand treatment of HAE attacks. Specifically, (1) How should “early” be defined when describing administration of on‐demand treatment? (2) What barriers exist to early treatment? (3) What are the current burdens surrounding use of on‐demand treatment, and what strategies could reduce these burdens on patients? (4) What are the concerns about tolerability and convenience of on‐demand treatment, and what might alleviate these difficulties? (5) What issues surround patient–clinician communication and patient education about on‐demand treatment? This Delphi initiative was designed to address these issues by reaching consensus among global experts on these key areas.

## 2 Methods

This 3‐round modified Delphi consensus process was conducted based on the ACcurate COnsensus Reporting Document (ACCORD) principles [37], from April 2024 to May 2025. A modified Delphi is a study in which the preliminary statements are drafted based on the findings of a literature review rather than initial statements from the panel [37].

### 2.1 Selection of Chairs and Panelists

Delphi co‐chairs were identified by KalVista Pharmaceuticals Inc. as leading experts in HAE, and panelists were confirmed by the co‐chairs. A third‐party administrator, Oxford PharmaGenesis Inc. (Wilmington, DE, USA), invited the 19 recommended panelists to participate in this Delphi initiative. This process is consistent with other Delphi consensus procedures sponsored by pharmaceutical companies [29, 31, 38, 39]; neutrality on the Delphi topic (i.e., in this initiative, existing and future on‐demand treatments for HAE attacks) was encouraged and expected in all communication with the co‐chairs and panelists. Invitations to panelists included the language, “This initiative is sponsored by KalVista Pharmaceuticals, but the company will not participate and will remain impartial.” Eligible panelists included those who (1) were licensed to practice medicine (or similar relevant qualification) in their geographic region; (2) had treated ≥ 10 patients with HAE per year over the last 5 years; and (3) had been a contributing author on published guidelines or research within the past 7 years.

Panelists and their responses were kept anonymous during the Delphi procedure. Identifying information was not visible to the team conducting the analysis of the survey responses, the co‐chairs, or other panel members. Of the 19 invited panelists, all agreed to participate, in addition to the two co‐chairs. Panelists practiced in Europe (*n* = 10), North America (*n* = 6), the Asia‐Pacific region (*n* = 2), and South America (*n* = 1).

### 2.2 Study Design

The study protocol for this modified Delphi initiative was drafted by the administrator (Oxford PharmaGenesis) and was approved by the co‐chairs before the study was initiated. The sponsor (KalVista Pharmaceuticals Inc.) had no role in developing the protocol or in the subsequent Delphi process. A review of guidelines for HAE was conducted by the administrator and provided to the co‐chairs and panelists to contextualize the study objectives.

**FIGURE 1:**
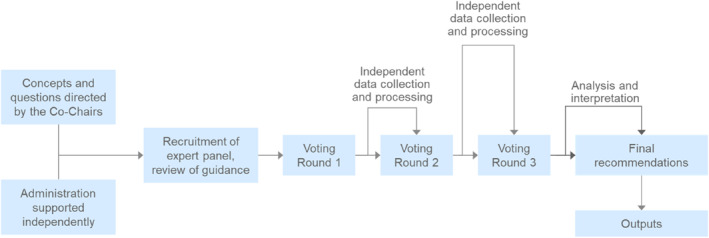
Delphi process.

The study format was prespecified in the protocol and designed according to Figure 1. The Delphi process was initiated by email to the panelists from the administrator describing the goals and responsibilities and included a brief pre‐survey about experience with HAE and contributions to HAE clinical guidelines. The panelists were also provided with a unique user ID#, a link to the review of the current guidelines, and a reference pack (hosted on the administrator's secure server). The unique user ID# was a required input on the questionnaire at each voting round for tracking purposes. The administrator kept this identification number secure and confidential, and it was not visible to the team conducting the analysis of the survey responses. No identifying information was shared with the co‐chairs or with other panelists, and respondents were de‐identified before analysis.

A detailed description of each round of statement development, voting, and analysis can be found in the Supporting Information S1. Briefly, panelists responded to three iterative surveys that were developed by the administrator based on direction and feedback from the co‐chairs. Following each survey, the results were analyzed, reviewed with the co‐chairs, and then sent to the panelists for their information. At the conclusion of Round 3, all statements that had reached ≥ 75% agreement were considered to be consensus statements.

### 2.3 Statistical Analysis

Mixed methods were used to analyze the qualitative and quantitative data collected during the Delphi process. A breakdown of the number of participants involved in each round was calculated, and descriptive summary statistics (i.e., frequency, percentages, mean) were calculated for categorical and continuous variables. Responses were grouped and tallied by frequency. Consensus was defined as ≥ 75% agreement with ≥ 75% of panel members voting. Degree of consensus was calculated as the percentage of votes with a score of ≥ 4 on a five‐point Likert scale.

## 3 Results

### 3.1 Summary

All 19 panelists participated in each of the three rounds of the Delphi consensus study. In Round 1, 13 open‐ended questions were provided to the panelists related to the five key areas (i.e., defining early administration, barriers to early treatment, burdens related to on‐demand treatment, on‐demand treatment tolerability and convenience, and patient–clinician interactions). Responses to these questions were categorized and used to develop 27 statements and three multiple‐choice questions for Round 2 voting. Of these, 19 statements reached consensus, though four of the 19 were refined with the aim of increasing the rate of consensus in Round 3. Eight statements did not reach consensus, and of those, four were refined for voting in Round 3. New statements were drafted based on the multiple‐choice questions, and two of those were voted on in Round 3.

During Round 3, 10 statements were presented to the panelists for voting. Sixteen statements that reached consensus in Round 2 and were not subsequently refined were presented to the panelists for their information. All 10 voting statements reached consensus. Discussion during the final presentation of results to the panelists and co‐chairs led to the removal of two statements. A total of 24 statements were approved as consensus statements.

### 3.2 Defining Early On‐Demand Treatment for HAE Attacks

Three statements related specifically to early on‐demand treatment for HAE reached the ≥ 75% threshold (Table 1). The panel agreed to define early treatment as 60 min or less after the onset of an HAE attack. Statements about treatment during the prodromal stages of an HAE attack and about administering on‐demand treatment even if the attack appears to be past the peak of severity did not reach consensus during Round 2 and were removed from subsequent voting.

**TABLE 1:** Consensus statements regarding defining early on‐demand treatment for HAE attacks.

Statement	Degree of consensus
Early on‐demand treatment should be initiated as soon as patients recognize any symptoms associated with an HAE attack	84%
Early treatment should be defined as 60 min or less after the onset of an HAE attack	95%
If the patient missed the opportunity to initiate early on‐demand treatment for an HAE attack, treatment should still be administered if the attack symptoms are progressing	84%

*Note:* Dark green indicates > 90% consensus and light green indicates > 80–90% consensus.

Abbreviation: HAE, hereditary angioedema.

### 3.3 Barriers to Early On‐Demand Treatment

Three statements related to the barriers patients face in administering early treatment for HAE attacks reached consensus (Table 2). The panel determined that barriers included inability to identify the onset of attacks, embarrassment about administering treatment, and potential delays caused by lack of assistance in administering treatment.

**TABLE 2:** Consensus statements regarding barriers to early on‐demand treatment for HAE attacks.

Statement	Degree of consensus
Patients sometimes struggle to identify onset of HAE attacks, especially in the case of mild or abdominal attacks, which may prevent them from administering early on‐demand treatment	95%
Some patients have embarrassment or anxiety about administering parenteral HAE on‐demand treatment, and that may prevent them from early treatment	84%
Administration of on‐demand HAE treatment can be delayed due to caregivers being unavailable, lack of HAE disease/treatment awareness of emergency medical staff, and long waiting times in emergency rooms	95%

*Note:* Dark green indicates > 90% consensus and light green indicates > 80–90% consensus.

Abbreviation: HAE, hereditary angioedema.

### 3.4 Burdens Related to On‐Demand Treatment

Six statements related to general burdens of administration (i.e., whether to treat) and access (i.e., cost, having enough doses at hand, approval of particular medications by region) of on‐demand treatment reached consensus (Table 3).

**TABLE 3:** Consensus statements regarding the burden of on‐demand treatment for HAE attacks.

Statement	Degree of consensus
Patients sometimes are unsure if an HAE attack is severe enough to warrant treatment, which may prevent them from administering on‐demand treatment	89%
Some patients lack the knowledge that all HAE attacks should be treated or choose not to treat attacks because previous attacks have resolved without treatment	89%
Some patients lack the training, ability, and/or confidence to self‐administer parenteral on‐demand HAE treatments, which may prevent them from administering on‐demand treatment	89%
Lack of access to on‐demand treatment for HAE attacks is a burden to patients	79%
The need for second or third doses of on‐demand treatment in some instances can be a burden for patients treating HAE attacks	84%
In some regions, the cost of on‐demand treatment for HAE attacks, including obtaining refills, can be a burden to patients and may prevent them from administering on‐demand treatment	89%

*Note:* Light green indicates > 80–90% consensus and yellow indicates ≥ 75–80% consensus.

Abbreviation: HAE, hereditary angioedema.

### 3.5 Burdens Related to the Tolerability and Convenience of On‐Demand Treatment

Across the six statements related to on‐demand treatment and tolerability, most panelists agreed that development of therapies that were easier and more convenient for patients to self‐administer could help alleviate some of the burdens associated with HAE (Table 4). During Round 1, some panelists raised concerns with the transportation of on‐demand medication and maintaining it at the correct temperature. Consequently, a statement for Round 2 was developed about potentially developing a medicine box to carry on‐demand medications; however, this statement did not reach consensus and was removed from the subsequent round.

**TABLE 4:** Consensus statements regarding treatment tolerability and convenience of on‐demand treatment for HAE attacks.

Statement	Degree of consensus
Side effects of parenteral on‐demand HAE treatments, including injection pain, can be an obstacle to early treatment, particularly in children	95%
Parenteral on‐demand HAE treatments present a variety of logistical burdens due to their route of administration, including transport and storage, and finding an appropriate place to self‐administer (i.e., clean, quiet, private)	95%
Improving HAE on‐demand options would reduce some of the burden associated with currently available on‐demand treatments for HAE attacks, including: Facilitating administration Facilitating use in younger patients Reducing the number and severity of side effects Increasing effectiveness	100%
On‐demand HAE therapies that have fewer side effects and are easier to store, carry, and administer may increase adherence to guidance on treating HAE attacks early	100%
Patients should have access to the on‐demand HAE treatment modality that they consider to be the most convenient	95%
Reducing the costs and increasing access/availability of on‐demand HAE treatments may increase patient adherence to guidelines regarding HAE attacks	100%

*Note:* Dark green indicates > 90% consensus.

Abbreviation: HAE, hereditary angioedema.

### 3.6 Patient–Physician Communication and Patient Education Regarding On‐Demand Treatment

The importance of patient–physician shared decision‐making and patient education regarding HAE treatment was emphasized across the six statements in this key area (Table 5). One statement about time constraints limiting the ability to utilize patient–physician shared decision‐making was included in the voting for Round 2, but did not reach consensus, and responses were too variable to refine the statement for further voting.

**TABLE 5:** Consensus statements regarding patient‐physician communication and patient education.

Statement	Degree of consensus
Patient–physician shared decision‐making should be utilized by physicians treating HAE	100%
The following items should be discussed during patient–physician shared decision‐making discussions for HAE, where relevant: Patient goals regarding activity, lifestyle, and quality of life, including travel and pregnancy plans Attack patterns, triggers, and severity The impact of not treating attacks All options for on‐demand therapy, including safety, efficacy, and routes of administration Options for prophylactic therapy and whether to use them Any barriers to initiating a new treatment Patient expectations of effectiveness and safety of the treatment Cost, access, and regulatory issues	100%
Patient behavior (infrequent visits or unwillingness to provide accurate feedback) can be a barrier to patient–physician shared decision‐making for HAE	95%
Some patients lack full understanding of HAE and/or awareness of the variety of treatment choices. These patients would benefit from regular, careful explanations in lay terms to establish trust and allow them an opportunity to make an informed decision	100%
Patients who lack the knowledge or confidence to self‐administer on‐demand HAE treatments would benefit from additional training	100%
Monitoring tools may help patients track their HAE attacks over time, understand symptoms, and develop criteria for identifying acute attacks to allow for timely on‐demand treatment administration	84%

*Note:* Dark green indicates > 90% consensus and light green indicates > 80–90% consensus.

Abbreviation: HAE, hereditary angioedema.

The final statements were formally endorsed by the co‐chairs and presented to the panelists.

## 4 Discussion

Multiple HAE organizations and study groups agree that HAE attacks should be treated as soon as possible with on‐demand therapy [8, 9, 26, 28, 29, 30, 31, 32, 33, 34, 35]. The goal of this Delphi consensus initiative was to gain a better understanding of the barriers to on‐demand treatment of HAE attacks. Therefore, key areas focused on: (1) defining early treatment, (2) barriers to early treatment, (3) describing burdens of on‐demand treatment, (4) detailing burdens related to tolerability and convenience of on‐demand treatments, and (5) clarifying patient–physician communication.

### 4.1 Defining Early On‐Demand Treatment for HAE Attacks

The initial pivotal studies of parenteral on‐demand treatments for HAE attacks, conducted from approximately 2005 to 2010, did not prioritize time from onset of attack to treatment administration, and in most cases, participants had to report to a study site for evaluation, delaying treatment compared with real‐world treatment patterns [12, 13, 40, 41, 42]. The IMPACT2 study of Berinert did not restrict the time from symptom onset to start of Berinert, and the median time between the estimated start of attack and treatment ranged from 3.2 to 5.9 h [12] Early studies of Ruconest also did not put a limit on the time from onset of attack to treatment, and did not collect data on this parameter [13]. In the pilot study of icatibant, patients were required to have arrived at the clinic within 10 h of the start of an attack. Among the 20 patients in this study, the time between onset of symptoms and start of treatment ranged from 1.9 to 10.3 h [40]. In the FAST‐3 study of icatibant, median time to treatment after attack onset was 6.5 h [41]. Moreover, many of these initial studies limited treatment to attacks that were deemed “moderate” or “severe,” [13, 40, 42] which may have delayed treatment, as patients could have waited for an attack to worsen before seeking treatment.

Subsequent research has shown that early treatment of HAE attacks can result in better treatment outcomes [41, 43, 44, 45, 46, 47, 48], and this approach is recommended by current treatment guidelines [8, 9]. Arresting the progression of swelling as early as possible after attack onset minimizes symptom burden and prevents disruption to a patient's life [8]. In a post hoc analysis of the IMPACT studies of C1‐INH concentrate, median time to complete resolution of HAE symptoms was shorter when treatment was administered within 6 h versus after 6 h after attack onset (IMPACT 1: 2.8 vs. 7.9 h; IMPACT 2: 12.6 vs. 14.4 h) [45]. Post hoc analyses of EDEMA ecallantide trials found that the greatest percentage of patients who experienced complete or near complete symptom resolution within 4 h after treatment (71.4%) were those who received ecallantide within 2 h of symptom onset [43]. In the Phase 3 FAST‐3 trial of icatibant, in which treatment had to be administered by a healthcare professional, median time from attack onset to treatment was 6.5 h, and median time to symptom resolution was 8.0 h; however, in subsequent real‐world analyses of patients who could self‐administer icatibant, median time from attack onset to treatment was 2.0 h and median time to symptom resolution was 3.5 h [41]. Furthermore, the Icatibant Outcome Study found that when patients were stratified by time to treatment, patients who treated their attacks in < 1 h had a significantly shorter median time to resolution (5.8 vs. 8.8 h) and attack duration (6.1 vs. 16.8 h) compared with patients who treated their attacks ≥ 1 h after onset [49].

However, while early on‐demand treatment provides a better treatment response than late treatment, and attacks treated earlier result in a shorter time to resolution of symptoms and shorter total attack duration, early treatment has not been well‐defined historically, and this lack of specificity can be challenging for patients who strive to adhere to guidelines. A 2024 survey of patients in the United States with HAE reported a mean time to treatment for the last attack of 3.8 h, even though two‐thirds of survey participants said they treated their attacks early after onset [44]; similar results were seen in an Italian survey in which 71% of respondents reported that they treated the attack early, but only 14% administered treatment < 1 h after attack onset, and the mean delay to treatment was 2.9 h [48]. In this Delphi consensus process, when asked to select an option for how early treatment should be defined, 18 of the 19 panelists selected either ≤ 30 min or ≤ 1 h, emphasizing the need for patients to treat their HAE attacks quickly after symptoms start. In some cases, patients are not able to treat immediately, and most panelists also agreed that in this scenario, they should still treat the attack if symptoms were progressing. Using the definition for early treatment of 60 min or less recommended by this Delphi consensus panel, clinicians can now provide a specific timeframe to their patients, and this guidance may help enable more prompt use of on‐demand treatment for HAE attacks.

Topics that did not reach consensus, such as treatment during the prodrome and treatment after an attack that appears to be past the peak of severity or decreasing in severity, demonstrated areas requiring additional data and could be considered as future topics in HAE research. Prodromes are defined as the signs and symptoms preceding an attack [50, 51]. Reviews of patient reports revealed that most patients experienced a prodrome before at least one attack, and 64% of patients were able to use their prodrome to predict an upcoming attack [50, 52]. A study published in 2025 administered recombinant human C1INH during the prodrome, and found that patients who were treated during the prodrome did not progress to an attack [51], but literature on this treatment strategy is sparse, resulting in lack of consensus on this topic. Multiple panelists mentioned treatment during the prodrome in the Round 1 survey, and a consensus statement was proposed for Round 2. While two panelists strongly agreed and seven panelists agreed that on‐demand treatment could be considered during the prodrome stages of an HAE attack, other panelists were doubtful about this course of action, leading to a score of 47%. Multiple panelists who provided comments indicated a lack of data for this approach, while others cited the inconsistency of the predictive value of prodromes as reasons for their disagreement with the statement. This issue arose again in Round 3 when two panelists thought the revised statement, “Early on‐demand treatment should be initiated as soon as patients recognize any symptoms associated with an HAE attack,” might be interpreted as recommending treatment during the prodrome and did not agree with that perceived recommendation. Although consensus about treating during the prodrome was not reached among the panelists, it remains a topic of interest in the HAE field, and patients should be encouraged to discuss it with their physicians. If a patient and their clinician agree that treatment during prodrome is in the patient's best interest, shared decision‐making is the right framework here.

Use of on‐demand treatment when attacks appear past peak severity was also a topic in which panelists differed in opinions and mentioned lack of available data. A Round 2 statement was proposed stating that on‐demand treatment should be administered in these circumstances, but only 47% of panelists agreed. While some panelists mentioned that attacks could migrate and were still worth treating, other panelists did not think treating past the peak of an attack would provide further or faster improvement, or did not think success was guaranteed. Still other panelists thought this should depend on severity or location of attack, or patient preference. Moreover, determining when an attack is at peak severity can be challenging for patients.

### 4.2 Barriers to Early On‐Demand Treatment

Panelists described multiple factors that could contribute to delayed on‐demand treatment of an HAE attack. Patients might not have on‐demand treatment readily available. They might be unable to identify the start of an attack. In some cases, patients are not able to self‐administer their on‐demand treatment and rely on caregivers or healthcare personnel, which could delay treatment, depending on availability. Panelists also noted that embarrassment or anxiety about administering parenteral treatment could be an obstacle to early treatment. Patient‐reported data support this statement; in a survey of patients in the HAE Association database, 32.5% of adults and 50.0% of adolescents described being extremely anxious about using on‐demand treatment for HAE attacks [44]. Reasons for anxiety varied by age, the most common reason in adults was availability and cost, and the most common source of HAE‐related anxiety in adolescents was finding a vein for administration [44].

### 4.3 Burdens Related to On‐Demand Treatment

Consensus statements also identified burdens related to on‐demand treatment for HAE attacks. Many of these statements were generally consistent with results from surveys of patients with HAE [44, 48], demonstrating that patients and clinicians have similar concerns. Determination of an attack's severity and lack of knowledge that all attacks should be considered for treatment were identified as barriers to self‐administration of treatment. Comments from panelists indicated that while they recommend that their patients consider treating all attacks, many patients hesitate to treat if they do not think the attack will be severe because of the financial costs of treatment or other burdens. This is consistent with published studies in which patients' survey responses described common barriers to treatment as uncertainty about whether the attack was real, belief the attack would be mild, and desire to save their on‐demand treatment for a severe attack [44, 48]. Moreover, previous guidelines had advised that patients only treat laryngeal or severe abdominal attacks [27], which could be a factor in hesitance to treat attacks. These factors add complexities for patients who struggle to make difficult decisions regarding early administration of on‐demand treatment. Panelists also noted that ability and/or confidence with self‐administration techniques of parenteral treatments could be a burden, potentially preventing patients from administering on‐demand treatment.

Many panelists described lack of access to on‐demand treatment as a burden in the free‐text responses in Round 1. However, attempts to further refine the specific causes for lack of access demonstrated the variability of access to treatment across geographical regions, making it challenging to define in this global Delphi panel. In Round 2, panelists were given a multiple‐choice question with the option to select specific factors that could lead to lack of access. In answering this question, 63% of panelists indicated that quantity limits for the number of doses obtainable over a set time period was a concern for their patients. Regulatory approval of certain therapies in the respondent's geographic region was a factor for 42% of panelists. A patient's ability to travel to a pharmacy to obtain medication was noted by 32% of panelists. The ability of patients to be reimbursed for certain therapies in the respondent's geographic region was selected by 26% of panelists. This range of responses was addressed in Round 3 by including a broad statement about lack of access being a barrier to on‐demand treatment. While this statement reached consensus, the rate of agreement was just above the threshold, with some panelists wanting to strengthen the statement (i.e., with language that lack of access can lead to disability, morbidity, and mortality risk), and other panelists wanting to qualify the statement to say that lack of access “can be” a burden or emphasizing that access can be influenced by geographic region and/or country. Discussing the impact of access to medication can also be challenging because “access” can refer to such diverse factors as physical access (i.e., having the medication on‐hand at the time of an attack), cost of the medication, insurance and reimbursement coverage, ability to obtain multiple doses and/or refills, and regulatory issues that impact what drugs are approved in different countries, all of which can be structural barriers that complicate patients' decision‐making regarding on‐demand treatment for HAE attacks.

### 4.4 Burdens Related to the Tolerability and Convenience of On‐Demand Treatment

Other obstacles to implementing treatment guidelines for on‐demand therapy for HAE attacks relate to tolerability and convenience of parenteral treatments, and the panelists considered a number of statements about these obstacles. Panelists noted that the side effects of certain on‐demand treatments, including injection pain, could be a barrier to treatment. A survey of real‐world treatment patterns included 94 patients with HAE who reported at least one attack in the 3 months before the survey; of the 77 who reported adverse events associated with their most recent treated attack, 44.2% listed the most severe adverse effect as “burning or pain while injecting the medication.” [44] This was acknowledged as a particular issue in children; as of the 12 adolescents included in the survey, 25.0% reported treatment‐related anxiety specific to “anticipating burning or pain with the injection.” [44] An Italian survey of patients with HAE also noted that adolescents were more likely than adults to delay treatment because of administration‐related barriers [48]. Other burdens, including cost of treatment, finding an appropriate place to administer a parenteral treatment, and the need for multiple doses, were also identified by the Delphi panel as reasons patients may delay treatment.

Most panelists agreed that patients should have access to the treatment modality that they considered the most convenient, and several panelists mentioned that medications with an easier route of administration could decrease the burden of treatment. Potential options mentioned were oral therapies (tablets, capsules, or sublingual treatments), transdermal treatments, or intranasal administration. While treatment modalities other than parenteral administration could be easier to carry and potentially be easier to self‐administer, some patients might have difficulty swallowing a pill, particularly if the patients are young, or if an attack involves the airway. Others might have skin sensitivity to a transdermal medication or struggle with intranasal administration. Providing additional options for on‐demand treatment beyond those available at the time of this Delphi consensus panel could potentially reduce the burden of treating attacks and improve adherence to guidelines on treating attacks early. However, an important caveat is that our consensus statements were focused on convenience, and safety and efficacy are crucial considerations that need to be prioritized.

A common theme across Round 1 responses was that patients might not carry treatment with them, and some panelists specifically mentioned concerns about the need to store medication at the appropriate temperature. Of the on‐demand treatments for HAE attacks that can be self‐administered by patients available as of June 2025, most should be stored at 36°F–77°F (2°C–25°C), though Berinert is stable up to 86°F (30°C) [10, 11, 16, 19]. For this reason, a statement about development of a medicine box to facilitate transporting on‐demand HAE treatments at the proper temperature was presented to the panelists, but did not reach consensus in Round 2 voting. Finally, all panelists agreed that reducing costs and increasing availability of on‐demand treatment for HAE attacks would improve patient adherence to treatment guidelines.

### 4.5 Patient–Physician Communication and Patient Education Regarding On‐Demand Treatment

The final key area focused on how patient–physician interactions and patient education could be optimized to help improve patients' knowledge about their HAE and use of on‐demand treatment. All panelists agreed that patient–physician shared decision‐making should be utilized. The panelists also developed a list of important items that should be discussed during shared decision‐making, and all agreed that careful explanations should be provided to patients who lack full awareness about HAE and/or its treatments. Prodromes and potentially administering on‐demand treatment during the prodrome can also be discussed as part of patient–physician shared decision‐making. Other statements focused on additional training on administration of on‐demand treatments, and encouraging patients to use resources (i.e., a diary [paper or electronic], questionnaire, scales for tracking symptoms) to better understand their symptoms. Multiple panelists agreed that these tools could be helpful, but mentioned the caveat that many patients do not use them consistently, limiting their value. Most panelists agreed that at times, patient behavior could be a barrier to optimal shared decision‐making, but one panelist cautioned against blaming patients, as patient behavior often has roots in previous unsatisfactory interactions with medical services.

When asked about time constraints during Round 2, 53% of panelists indicated that factors, such as number of patients per day, amount of time during an office visit, and frequency of visits, limited their ability to facilitate patient–physician shared decision‐making in HAE. These issues could be related to the geographic region in which panelists practice. Healthcare laws and regulations could impact the time clinicians spend with individual patients in certain regions, which could also be related to the types of institutions where the panelists primarily practice. Private practice, hospitals, or government‐run facilities could all have different requirements or demands on the time of healthcare professionals.

### 4.6 Limitations

While Delphi consensus procedures offer a valuable method to compile feedback from a range of experts, there is little consistency in the way these procedures are executed, making design and analysis challenging [53]. However, important attributes have been identified, and this Delphi attempted to address each of these: anonymity of panelists, a moderator to review feedback, iterative rounds of questions, predefined consensus criteria and closing criteria, and three rounds of review. Delphis are often conducted in situations where there is a lack of supporting data, as is frequently the case in rare diseases. For this reason, many of the statements developed during this iterative process are based on expert opinion rather than clinical data. Although panelists in this study did not differentiate between laryngeal attacks and those occurring in other locations, it is important to note that laryngeal attacks can be life‐threatening and progress rapidly, they represent a unique emergency scenario and might justify even stronger emphasis on immediate therapy initiation.

This Delphi panel spanned multiple countries across multiple regions, and while the breadth of geographic regions is an asset at presenting a wide range of perspectives, it can also complicate interpretation of results surrounding access, cost, and insurance. These factors are more dependent on healthcare systems and regulatory policy than clinical aspects, and thus vary considerably depending on geographic region, making it difficult to reach consensus. While there was diversity across the panelists regarding geographic location, all panelists were clinicians, by study design, and perspectives from patients or advocacy groups were not included in developing the consensus statements. The Delphi consensus process relies on anonymity among panelists, but for rare diseases, such as HAE, the field is small, and most panelists likely knew at least some of their fellow panelists and how they might respond to certain questions. Anonymity also prevented the panelists from discussing the statements among themselves, though this was mitigated by the review and refinement of the results by the co‐chairs.

Finally, this Delphi was conducted during a time of transition in the treatment of HAE attacks. At the time the study was conducted, the only approved on‐demand treatments for HAE attacks were parenteral. However, many of the panelists were aware of oral therapies in late‐stage development, which could have impacted their responses. At the time of the study, investigational oral therapies included sebetralstat (EKTERLY, KalVista Pharmaceuticals), which had positive results in Phase 2 and 3 testing [24, 54] and was approved by the US FDA in July 2025 [21] (after completion of this Delphi study) and subsequently in the European Union and the United Kingdom [22], and deucrictibant (Pharvaris), which has completed Phase 2 for on‐demand treatment of HAE attacks [25].

### 4.7 Conclusion

Within this Delphi process, consensus was generally high among the 19 panelists. A total of 24 statements about issues of the use of on‐demand treatment for HAE attacks were developed, notably, defining early treatment of HAE attacks as 60 min or less after the onset of an attack. Statements spanned five key areas: addressing the definition of early administration of treatment, barriers to early treatment, burdens of treatment, tolerability and convenience of treatment, and patient–physician communication about HAE. The statements agreed on by the panel demonstrate a need in this population for accessible and convenient on‐demand treatments that will allow patients to improve adherence to HAE guidelines.

## Author Contributions

**Aleena Banerji:** conceptualization, investigation, methodology, project administration, supervision, validation, writing – original draft, writing – review and editing. **Emel Aygören‐Pürsün:** investigation, methodology, writing – original draft, writing – review and editing. **Noemi‐Anna Bara:** investigation, methodology, writing – original draft, writing – review and editing. **Jonathan A. Bernstein:** investigation, methodology, writing – original draft, writing – review and editing. **Stephen Betschel:** investigation, methodology, writing – original draft, writing – review and editing. **Laurence Bouillet:** investigation, methodology, writing – original draft, writing – review and editing. **Paula J. Busse:** investigation, methodology, writing – original draft, writing – review and editing. **Teresa Caballero:** investigation, methodology, writing – original draft, writing – review and editing. **Mauro Cancian:** investigation, methodology, writing – original draft, writing – review and editing. **Danny M. Cohn:** investigation, methodology, writing – original draft, writing – review and editing. **Timothy Craig:** investigation, methodology, writing – original draft, writing – review and editing. **Henriette Farkas:** investigation, methodology, writing – original draft, writing – review and editing. **Anete Sevciovic Grumach:** investigation, methodology, writing – original draft, writing – review and editing. **Michihiro Hide:** investigation, methodology, writing – original draft, writing – review and editing. **Sorena Kiani‐Alikhan:** investigation, methodology, writing – original draft, writing – review and editing. **Hilary J. Longhurst:** investigation, methodology, writing – original draft, writing – review and editing. **William R. Lumry:** investigation, methodology, writing – original draft, writing – review and editing. **Marc A. Riedl:** investigation, methodology, writing – original draft, writing – review and editing. **Marcin Stobiecki:** investigation, methodology, writing – original draft, writing – review and editing. **Anna Valerieva:** investigation, methodology, writing – original draft, writing – review and editing. **Andrea Zanichelli:** conceptualization, investigation, methodology, project administration, supervision, validation, writing – original draft, writing – review and editing.

## Funding

We thank the participants involved in this Delphi study. Panelists were not paid an honorarium for their contributions to the Delphi study or any ensuing publication activities. KalVista Pharmaceuticals Inc. provided an unrestricted grant to fund the study, with no involvement beyond funding. Third‐party administrative and project management support was provided by Oxford PharmaGenesis Inc., Wilmington, DE, USA. Medical writing support was provided by Heather A. Mitchell, PhD, of Oxford PharmaGenesis Inc., Wilmington, DE, USA, and was funded by KalVista Pharmaceuticals Inc.

## Conflicts of Interest

Aleena Banerji has received support for research from Ionis, Astria, and Intellia; and for consulting from CSL, Ionis, Pharvaris, Intellia, KalVista, Astria, ADARx, and BioCryst.

Emel Aygören‐Pürsün has received grants, consulting fees, honoraria, fees paid to the institution, and/or personal fees from KalVista, Astria, BioCryst, CSL Behring, Intellia, Otsuka, Pharvaris, and Takeda/Shire.

Noemi‐Anna Bara has received honoraria for educational lectures, consultancy, sponsorship for educational meetings, research projects from Takeda/Shire, Pharming Group N. V., CSL Behring, Pharvaris, KalVista, and BioCryst.

Jonathan A. Bernstein reports support as a PI and consultant for Takeda/Shire, CSL Behring, Pharming, BioCryst, KalVista, Ionis, Astria, Intellia, Pharvaris, ADARx; speaker fees from Pharming, KalVista, CSL Behring; HAEA MAB, WAO BOD, AAAAI Foundation, and ACARE/UCARE COE.

Stephen Betschel reports grants or contracts from Astria, BioCryst, CSL, Ionis, KalVista, Pharvaris, and Takeda; consulting fees from Astria, BioCryst, CSL, KalVista, and Takeda; payment or honoraria for lectures, presentations, speaker's bureaus, manuscript writing or educational events from BioCryst, KalVista, CSL, and Takeda; support for attending meetings and/or travel from Astria, BioCryst, KalVista, CSL, and Takeda; participation on a data safety monitoring board or advisory board from Ionis, Takeda, and Pharvaris; and is the Chair of the Canadian Hereditary Angioedema network.

Laurence Bouillet has consulted/served as speaker for, engaged in research and educational projects with, or accepted travel grants from the following companies: BioCryst, CSL Behring, Takeda, KalVista, Pharvaris, and Otsuka.

Paula J. Busse has received consulting fees and/or research grants/contracts from ADARx, Astria, BioCryst, CSL Behring, CVS Specialty, Intellia, KalVista, Pharvaris, and Takeda; and has served as medical advisor for the Hereditary Angioedema Association (HAEA).

Teresa Caballero reports research funding from AEDAF, CSL Behring, Takeda; speaker honoraria from BioCryst, CSL Behring, Novartis, Organon, Takeda; consulting honoraria from Astria, BioCryst, CSL Behring, Ionis, KalVista, Novartis, Otsuka, Pharvaris, Takeda; funding for scientific meeting attendance from BioCryst, CSL Behring, Novartis, Takeda; is a researcher in clinical studies/registries/clinical trials for BioCryst, CSL Behring, Ionis, KalVista, Novartis, Takeda; and has received funding for manuscript writing and publication from BioCryst, KalVista, and Takeda.

Mauro Cancian has received grant research support and/or speaker/consultancy fees from BioCryst, Chiesi, CSL Behring, KalVista, Novartis, Otsuka, Pharming, Pharvaris, Sanofi, and Takeda.

Danny M. Cohn has received consulting fees paid to the institution, honoraria paid to the institution, meeting/travel support, and research support; has served on advisory boards for KalVista, Astria, BioCryst, CSL Behring, Intellia, Ionis, Otsuka, Pharvaris, and Takeda; and has a leadership role for the HAE International (HAEi) medical advisory panel for Central Eastern Europe and Benelux.

Timothy Craig received research support and was a consultant for CSL Behring, Ionis, Takeda, BioCryst, BioMarin, KalVista, Pharvaris, Intellia, ADARx, and Astria; received speaker fees from CSL Behring, Astria, KalVista, and Takeda; and travel support from CSL Behring, Takeda, and BioCryst.

Henriette Farkas has received grants paid to the institution, honoraria, meeting/travel support, and/or served on advisory boards for KalVista, Astria, BioCryst, CSL Behring, Intellia, Ono Pharmaceutical, Pharming, Pharvaris, and Takeda; and has served in a leadership role on the Angioedema Centers of Reference and Excellence (ACARE) Steering Committee.

Anete Sevciovic Grumach receives research funding from the Brazilian National Council for Scientific and Technological Development (CNPq) and received a grant of researcher initiative from Takeda/Shire; and is or recently was a speaker and/or advisor for Catalyst Pharmaceuticals, CSL Behring, Takeda, KalVista, Pharvaris, Pint‐Pharma, MultiCare, and Astria.

Michihiro Hide has received speaker/consultancy fees from Astria, CSL Behring, KalVista, Pharvaris, Takeda, and Torii.

Sorena Kiani‐Alikhan is a chief and/or principal investigator for studies and in receipt of honoraria for consulting work and advisory boards organized by: Takeda/Shire, CSL Behring, BioCryst, Biotest, KalVista, Pharvaris, X4 Pharmaceuticals, Ionis, Astria, and Otsuka.

Hilary J. Longhurst has participated in research and/or educational initiatives and/or served as advisor or speaker for the following companies: Astria, CSL Behring, Intellia, KalVista, Pharvaris, and Takeda within the past 3 years.

William R. Lumry is a member of advisory boards for BioCryst, CSL Behring, and Takeda; has received research grants from BioCryst, CSL Behring, Ionis, and Takeda; consulting fees from BioCryst, CSL Behring, Fresenius Kabi, Pharming, and Takeda; payments for lectures from CSL Behring, Pharming, and Takeda; and is an advisory board member of the US Hereditary Angioedema Association.

Marc A. Riedl is or recently was a speaker and/or advisor for and/or has received research funding from Astria, BioCryst, BioMarin, Celldex, CSL Behring, Cycle Pharma, Grifols, Intellia, Ionis, KalVista, Pfizer, Pharming, Pharvaris, Sanofi‐Regeneron, and Takeda.

Marcin Stobiecki has received honoraria for educational lectures, consultancy, sponsorship for educational meetings, research projects from CSL Behring, Ionis, KalVista, Pharming, Pharvaris, and Takeda.

Anna Valerieva has received honoraria for educational lectures, consultancy, sponsorship for educational meetings, research projects from Takeda/Shire, Pharming Group N. V., CSL Behring, SOBI, AstraZeneca, Berlin‐Chemie/Menarini Group, Teva, Novartis, Ewopharma, Stallergenes Greer, Pharvaris, KalVista, Ionis, Astria, and Organon.

Andrea Zanichelli has received honoraria, meeting/travel support, and/or served on advisory boards for KalVista, Astria, BioCryst, CSL Behring, Pharming, Pharvaris, and Takeda.

## Supporting information


Supporting Information S1

## Data Availability

The original contributions presented in the study are included in the article and Supporting Information S1. Further inquiries can be directed to the corresponding author.
