# Supplementary material for: An International Delphi Study on Barriers to On‐Demand Treatment of Hereditary Angioedema Attacks
Source: Clin Transl Allergy. 2026 Mar 4;16(3):e70159. doi: 10.1002/clt2.70159 (PMC12960019; doi:10.1002/clt2.70159)
Supplement: Supplementary file 1 — Supporting Information S1 [file CLT2-16-e70159-s001.docx]

# SUPPLEMENTAL MATERIAL

# Supplemental methods

## Round 1

Questions for Round 1 were developed by the co-chairs, with support from the administrator. The Round 1 questionnaire consisted of 13 total questions; 10 free-text and 3 that requested panelists respond on a 5-point Likert scale. The Likert scale asked respondents to rank a statement (or individual items) on a scale from 1 to 5.

Questions focused on the 5 key areas (ie, defining early treatment, barriers to early on-demand treatment, the burdens associated with on-demand treatment, tolerability and convenience burdens with on-demand treatment, and patient-physician communication). The questionnaire was sent to panelists via Survey Monkey, and panelists were given 2 weeks to complete it. Nonresponders were contacted a minimum of 2 times with reminders by email or telephone. Responses from a minimum of 10 panel members were required at each voting phase, and participation in the prior round was required for participation in each subsequent round.

Open-ended free-text questions were analyzed qualitatively to identify common themes for development of consensus statements. Verbatim responses were screened to identify common themes for each question, and responses from each panelist were assessed to quantify which themes appeared in each answer. Answers from an individual panelist could contain multiple themes. Responses were grouped and tallied by frequency. Likert scale questions were analyzed quantitatively to describe the number of panelists who responded with which value. The results were presented to the co-chairs, who assisted in development of questions and consensus statements for Round 2.

## Round 2

The Round 2 survey was routed to the panelists via Survey Monkey, and panelists were asked to rate the degree to which they agreed or disagreed with each of the 27 statements on the Likert scale. Each statement also had a free-text space to provide additional information. In addition to the statements, Round 2 included 3 questions on which panelists could choose either one or multiple options.

Responses were assessed for consensus and results were presented to the co-chairs, who provided feedback for the refinement of statements for Round 3. Likert-scale statements were analyzed quantitatively to determine the level of consensus for each statement. Responses of “strongly agree” and “agree” were considered agreement, and responses of “neither agree nor disagree,” “disagree,” and “strongly disagree” were considered lack of agreement. To reach consensus, statements required ≥75% agreement, with ≥75% of panelists responding. Free-text comments on each question were reviewed to inform refinement of the statements.

Statements were confirmed, revised, or removed from analysis based on degree of consensus and free-text feedback from the panelists, in addition to direction from the co-chairs. If a statement reached consensus with no or minimal editorial-only feedback for revision, it was considered confirmed. If a statement reached consensus but also had actionable feedback for revisions that were expected to increase the rate of agreement, the statement was refined and included in the voting for Round 3. If a statement did not reach consensus, but panelists had provided feedback for revision of the statement, the statement was refined and included in the voting for Round 3. If a statement did not reach consensus, without actionable feedback for revision, it was removed from analysis.

Multiple-choice questions were analyzed based on the degree of consensus achieved for each possible response. For the question where panelists were permitted to select a single option, responses were considered individually to develop a new statement for voting in Round 3. For the questions where panelists could choose multiple options, each selection that reached ≥75% agreement was incorporated into a new consensus statement for Round 3 voting.

## Round 3

The Round 3 survey was based on the results of Round 2, with input from the co-chairs. Any statements that had achieved consensus and not required any refinement were considered confirmed and not included in the Round 3 survey, but were presented to the panelists with free-text boxes, so that panelists could provide feedback if they wished to do so. In Round 3, panelists voted on Likert-scale statements that had been developed based on the multiple-choice questions and statements that had been refined following feedback in Round 2. These statements were analyzed by the same methods as the Likert-scale statements in Round 2, with the same ≥75% threshold for consensus.

The final version of the statements was presented to the Delphi consensus panel.

# Supplemental data

## Supplemental Table 1. Guidelines reviewed

| **Organization** | **Year** | **Citation** |
| --- | --- | --- |
| WAO/EAACI | 2022 | Maurer 2022^†^ |
| US HAEA | 2020 | Busse 2020^‡^ |
| Norma Clínica da Direção-Geral da Saúde 009/2019, 2019 PT DGS | 2019 | Branco Ferreira 2023 (described in English)^§^ |
| Canadian HAE Network | 2019 | Betschel 2019^¶^ |
| The Spanish Study Group on Bradykinin-Induced Angioedema, a working group of the Spanish Society of Allergology and Clinical Immunology | 2011 | Caballero 2011^††^ |
| HAE Society of India | 2023 | Jindal 2023^‡‡^ |
| German consensus on pediatric HAE | 2020 | Wahn 2020^§§^ |
| German Association of Scientific Medical Societies | 2019 | Bork 2019^¶¶^ |
| 9th C1 Inhibitor Deficiency Workshop in Budapest (panel meeting) | 2015 | Farkas 2017^†††^ |
| US HAEA | 2016 | Frank 2016^‡‡‡^ |

DGS, Direção-Geral de Saúde; HAE, hereditary angioedema; US HAEA, United States Hereditary Angioedema Association; WAO/EAACI, World Allergy Organization/European Academy of Allergy and Clinical Immunology.

# Supplemental references

^†^Maurer M, Magerl M, Betschel S, et al. The international WAO/EAACI guideline for the management of hereditary angioedema-The 2021 revision and update. *Allergy.* 2022;77(7):1961-1990.

^‡^Busse PJ, Christiansen SC, Riedl MA, et al. US HAEA Medical Advisory Board 2020 Guidelines for the Management of Hereditary Angioedema. *J Allergy Clin Immunol Pract.* 2021;9(1):132-150 e133.

^§^Branco Ferreira M, Baeza ML, Spinola Santos A, et al. Evolution of Guidelines for the Management of Hereditary Angioedema due to C1 Inhibitor Deficiency. J Investig Allergol Clin Immunol 2023;33:332-62.

^¶^Betschel S, Badiou J, Binkley K, et al. The International/Canadian Hereditary Angioedema Guideline. Allergy Asthma Clin Immunol 2019;15:72.

^††^Caballero T, Lleonart-Bellfill R, Pedrosa M, Ferrer L, Guilarte M. Expert Review and Consensus on the Treat-to-Target Management of Hereditary Angioedema: From Scientific Evidence to Clinical Practice. J Investig Allergol Clin Immunol 2023;33:238-49.

^‡‡^Jindal AK, Sil A, Aggarwal R, et al. Management of hereditary angioedema in resource-constrained settings: A consensus statement from Indian subcontinent. Asia Pac Allergy 2023;13:60-5.

^§§^Wahn V, Aberer W, Aygoren-Pursun E, et al. Hereditary angioedema in children and adolescents - A consensus update on therapeutic strategies for German-speaking countries. Pediatr Allergy Immunol 2020;31:974-89.

^¶¶^Bork K, Aygören-Pürsün E, Bas M, et al. Guideline: Hereditary angioedema due to C1 inhibitor deficiency. Allergo Journal International 2019;28:16-29.

^†††^Farkas H, Martinez-Saguer I, Bork K, et al. International consensus on the diagnosis and management of pediatric patients with hereditary angioedema with C1 inhibitor deficiency. Allergy 2017;72:300-13.

^‡‡‡^Frank MM, Zuraw B, Banerji A, et al. Management of Children With Hereditary Angioedema Due to C1 Inhibitor Deficiency. Pediatrics 2016;138.
